# Accuracy of intact performance on measures of odor identification, global cognition, and amyloid imaging for identifying lack of cognitive decline and dementia in the Mayo Clinic Study of Aging cohort

**DOI:** 10.1002/alz.088601

**Published:** 2025-01-03

**Authors:** Jeffrey N Motter, Seonjoo Lee, David S. Knopman, Maria Vassilaki, Davangere Devanand

**Affiliations:** ^1^ Columbia University Irving Medical Center, New York, NY USA; ^2^ New York State Psychiatric Institute, New York, NY USA; ^3^ Cognitive Neuroscience Division, Columbia University, New York, NY USA; ^4^ Mayo Clinic, Rochester, MN USA

## Abstract

**Background:**

Previous studies have linked impaired odor identification and global cognition with increased risk of cognitive decline and transition to dementia. However, the reverse question remains: if individuals have intact performance on these measures, are they at reduced risk for transition? We aimed to examine the accuracy of intact odor identification and global cognition for identifying lack of transition to dementia/cognitive decline using the population‐based Mayo Clinic Study of Aging and compare their accuracy against and in combination with amyloid PET (Positron Emission Tomography).

**Method:**

n = 647 participants age≥55 without dementia completed at baseline the Brief Smell Identification Test (BSIT; ‘Intact’ = 9‐12), Blessed Information‐Memory‐Concentration Test (BIMCT; ‘Intact’ = 18‐20,), and amyloid PET (‘Normal/Intact’ SUVR<1.48). We calculated sensitivity, specificity, positive predictive value, and negative predictive value for these measures, with “lack of transition to dementia” as the gold standard/target. “Lack of cognitive decline” (cognitively unimpaired to mild cognitive impairment [MCI]; cognitively unimpaired to dementia; or MCI to dementia) was a secondary target.

**Result:**

94.7% of participants (613/647) did not transition to dementia and 84.2% (545/647) did not cognitively decline over 11.25‐year‐follow‐up. The combination of intact BSIT+BIMCT had high rates of lack of transition to dementia (98.3%, 355/361) and lack of cognitive decline (93.1%, 336/361). This combination yielded mixed sensitivity (0.579) and specificity (0.824) though had high positive predictive value (0.983) for identifying lack of transition to dementia. Intact amyloid PET alone exhibited comparable rates of lack of transition to dementia (99.0%, 418/423) and lack of cognitive decline (92.4%, 391/423). When evaluated conjointly, the combination of all three intact measures also had mixed sensitivity (0.421) and specificity (0.962) though higher positive predictive value (PPV = 0.996) for lack of transition to dementia. 99.6% (258/259) of participants with intact/normal status on all three did not transition to dementia and 96.5% (250/259) did not cognitively decline.

**Conclusion:**

This replicates prior reports that intact olfaction/global cognition have strong utility for identifying individuals unlikely to develop MCI/dementia. Intact/normal amyloid PET further enhances the positive predictive value. Clinically, identifying individuals at low risk of transition can shorten diagnostic workups, reduce need for early follow‐up visits, and screen patients in‐or‐out of clinical trials.

